# Nanoparticle theranostics in cardiovascular inflammation

**DOI:** 10.1016/j.smim.2021.101536

**Published:** 2021-08

**Authors:** Neil MacRitchie, Valentina Di Francesco, Miguel Filipe Moreira Marques Ferreira, Tomasz J. Guzik, Paolo Decuzzi, Pasquale Maffia

**Affiliations:** a Centre for Immunobiology, Institute of Infection, Immunity and Inflammation, College of Medical, Veterinary and Life Sciences, University of Glasgow, Glasgow, United Kingdom; b Laboratory of Nanotechnology for Precision Medicine, Fondazione Istituto Italiano di Tecnologia, Genoa, Italy; c Institute of Cardiovascular and Medical Sciences, College of Medical, Veterinary and Life Sciences, University of Glasgow, Glasgow, United Kingdom; d Department of Internal Medicine, Jagiellonian University, Collegium Medicum, Kraków, Poland; e Department of Pharmacy, School of Medicine and Surgery, University of Naples Federico II, Naples, Italy

**Keywords:** ^99m^Tc, ^99^m-technetium, AAA, abdominal aortic aneurysm, apoE^−/−^, apoloporotein-E, CANTOS, Canakinumab Anti-inflammatory Thrombosis Outcomes Study, CCR5, C-C chemokine receptor type 5, CVD, cardiovascular diseases, CIRT, Cardiovascular Inflammation Reduction Trial, CITE-seq, cellular indexing of transcriptomes and epitopes by sequencing, COLCOT, Colchicine Cardiovascular Outcomes Trial, CT, computed tomography, CyTOF, cytometry by time of flight, DDS, drug delivery systems, DCE-MRI, dynamic contrast enhanced MRI, EPR, enhanced permeability and retention, FN-EDB, extra domain B of fibronectin, FDA, Food and Drug Administration, Gd^3+^, gadolinium, HDL, high-density lipoprotein, HDL-MNS, high-density lipoprotein-like magnetic nanostructures, hs-CRP, high-sensitivity C-reactive protein, H_2_O_2_, hydrogen peroxide, ICAM-1, intercellular adhesion molecule-1, IL-6, interleukin-6, IPAI, intravascular photoacoustic imaging, IVUS, intravascular ultrasound, LoDoCo2, low-dose colchicine 2, MPI, magnetic particle imaging, MRI, magnetic resonance imaging, MACE, major adverse cardiac events, MMPs, matrix metalloproteinases, MTX, mitoxantrone, MSOT, multispectral optoacoustic tomography, MPO, myeloperoxidase, MI, myocardial infarction, NP, nanoparticle, NPRC, natriuretic peptide receptor C, NIR, near-infrared, PFCs, perfluorocarbons, PAI, photoacoustic imaging, PDT, photodynamic therapy, PTT, photothermal therapy, PLGA, poly(lactic acid), poly(lactic-co-glycolic acid), PEG, polyethylene glycol, PET, positron Emission Tomography, PLP, prednisolone phosphate, PCSK9, proprotein convertase subtilisin-kexin type 9, PGI2, prostacycline, QD, quantum dots, RAGE, receptor for advanced glycation end-products, RES, reticuloendothelial system, SR-A, scavenger receptor-A, scRNA-seq, single-cell RNA sequencing, SPECT, single-photon emission computed tomography, SPR, surface plasmon resonance, SERS, surface-enhanced Raman spectroscopy, USPIO, ultra-small superparamagnetic iron oxide, VCAM-1, vascular cell adhesion molecule 1, Atherosclerosis, Cardiovascular disease, Inflammation, Myocardial infarction, Nanoparticles, Theranostics

## Abstract

Theranostics, literally derived from the combination of the words diagnostics and therapy, is an emerging field of clinical and preclinical research, where contrast agents, drugs and diagnostic techniques are combined to simultaneously diagnose and treat pathologies. Nanoparticles are extensively employed in theranostics due to their potential to target specific organs and their multifunctional capacity. In this review, we will discuss the current state of theranostic nanomedicine, providing key examples of its application in the imaging and treatment of cardiovascular inflammation.

## 1 Introduction

### 1.1 The role of inflammation in cardiovascular diseases

Atherosclerosis, which is the principal underlying pathology leading to myocardial infarction (MI) and stroke, is now recognised to be a disease both of hyperlipidaemia and chronic inflammation [1]. Extensive use of animal models has revealed the importance of both the innate and adaptive immune system in modulating atherosclerosis formation [2,3]. In particular, Ly6C^hi^ -monocyte derived macrophages are abundant in the growing plaque, where they promote a local pro-inflammatory environment in response to cholesterol accumulation, inflammasome activation and impaired efferocytosis [4]. The development of advanced immunophenotyping techniques such as cytometry by time of flight (CyTOF), single-cell RNA sequencing (scRNA-seq) and cellular indexing of transcriptomes and epitopes by sequencing (CITE-seq) are becoming important tools for interrogating the inflammatory environment in both animal models and human CVD patients [5] and may help reveal the most opportunistic therapeutic targets.

CVD patients frequently present with elevated circulating inflammatory markers including interleukin-6 (IL-6) and high-sensitivity C-reactive protein (hs-CRP) [6]. Indeed, despite aggressive treatment of hypercholesterolaemia using statins and proprotein convertase subtilisin-kexin type 9 (PCSK9) inhibitors, over one third of patients still have hs-CRP levels > 3 mg/L which is associated with an increased risk of future major adverse cardiac events (MACE) [7]. The acceptance that atherosclerosis is inherently associated with inflammation led to the Canakinumab Anti-inflammatory Thrombosis Outcomes Study (CANTOS) [8] and the Cardiovascular Inflammation Reduction Trial (CIRT) [9]. While the CIRT, utilising methotrexate, failed to reduce MACE, the CANTOS trial utilising canakinumab, a neutralizing anti-interleukin-1β (Il-1β) antibody, did reduce the incidence of recurrent MACE with respect to the placebo group. Importantly, patients in the CANTOS trial were selected based on residual inflammatory risk (hs-CRP >2 mg/L) whereas this was not the case in the CIRT. This resulted in patients in the CANTOS trial having a higher mean hs-CRP level (4.2 mg/L) compared with patients recruited to the CIRT (1.6 mg/L), a factor that may explain the lack of effect that methotrexate had in that study. Further clinical trials have employed colchicine in patients following MI (Colchicine Cardiovascular Outcomes Trial (COLCOT) [10] or in patients with chronic coronary artery disease (low-dose colchicine 2 (LoDoCo2) trial) [11]. In both trials, a significant reduction in composite MACE were shown in the treatment group. These results clearly demonstrate that anti-inflammatory agents can impact CVD. However, the long-term use of systemic anti-inflammatories can also pose a problem in terms of wider immune-suppression. Indeed, more fatal infections were observed in the CANTOS trial and a higher rate of non-basal skin cancer was observed in the CIRT study. One potential solution to circumvent these adverse events is targeted immunotherapy, utilising nanomaterials to deliver anti-inflammatory drugs directly to the site of pathology, allowing a higher concentration to be achieved while minimising the risk of systemic toxicity [12]. Nanomaterials are an active area of research and offer high versatility in both composition and function [13]. In addition to acting as drug carriers, nanomaterials are used both clinically and pre-clinically as diagnostic imaging agents [14]. Theranostics is the combination of drug delivery and imaging (and thus monitoring) the effect a drug has at the site of action. This review will outline the current state of theranostics with respect to inflammation in CVD.

### 1.2 Nanoparticle properties

Nanomedicine is a promising strategy to improve the diagnosis and treatment of cardiovascular diseases (CVD). Nanoparticles (NPs) offer unique features like the small size allowing easy uptake by the cells, the high surface-area-to-volume ratio, which controls the absorptions and sustained release of drugs, targeting features that can promote their preferential accumulation in specific sites [15]. Furthermore, the most important properties of nanoparticles are biocompatibility and biodegradability. In particular, the degradability of the NP can be an important property that ideally allows controlling the release of the cargo in the target site while remaining stable at off-target sites [16].

NPs are classified according to their size, shape, and material properties. Some classifications divide them between organic and inorganic NPs, with the first group including liposomes, polymeric nanoparticles, and polymeric nanoparticles, while the second group includes gold nanoparticles, quantum dots, and fullerenes. Other classifications divide NPs depending on their composition, whether they are carbon-based, ceramic, semiconducting, or polymeric. NPs can also be classified due to their stiffness as hard (silica particles, titanium dioxide and fullerenes) or soft (vesicles, liposomes and nanodroplets). Other classifications divide them based on their application, for example, the ones used “in diagnosis or therapy” *vs*. the ones used in “basic research”.

The physical characteristics of NPs can differ in many ways [17]. In particular:

Size: NPs are defined as nanomaterials having all three dimensions in the 1−100 nm size range, even if other dimensions outside this range are also considered [18]. The smaller size allows reaching novel structural form and new optical and electronic properties, improves NP solubility and increases NP bioavailability and circulation time;

Shape: NPs can be spheres, discs, hemispheres, cylinders, cones, tubes, or wires. Different shapes allow different interactivity, loading capacity, and transport capabilities;

Surface area: as particle size decreases, the total surface area increases exponentially, that means that a greater portion of atoms is located on the surface of the particles relative to the core. This makes NPs more reactive and more prone to be conjugated with electrostatic charges or biomolecules selected for targeting or other purposes;

Permeability: NP small size can facilitate the crossing of biological barriers that are normally not accessible.

Following administration of NP (most commonly via intravenous injection), they may accumulate solely through the enhanced permeability and retention (EPR) effect, a process referred to as passive uptake and is particularly prominent in the context of increased vascularisation or endothelial permeability, such as occurs in tumours or sites of active inflammation. Conversely, NP can be functionalised by incorporating targeting ligands such as antibodies or peptides on the NP surface directed against disease-specific marker(s) (active targeting). In addition to targeting ligands, NP can incorporate imaging agents and/or therapeutic payloads, either attached to the NP surface or encapsulated inside. The large surface area to volume ratio of NPs allows significant flexibility in surface modifications and presents a large area for immune cell recognition and adsorption of endogenous biomolecules. In fact, during the circulation in the bloodstream, NPs are easily recognised and phagocytosed by macrophages because they are bind by opsonins (immunoglobulins and C3, C4 and C5 complement proteins). For these reasons, NPs are often coated with a hydrophilic polymer such as polyethylene glycol (PEG) or zwitterionic polymeric forms to prolong residence times and reduce clearance by the reticuloendothelial system (RES) [19,20]. Also, the physical properties of the NP can be modulated to avoid rapid clearance. For example, NPs should be larger than 10 nm to avoid renal filtration passing through the fenestrated capillary endothelium of the glomerulus [21]. At the same time, particles with a diameter greater than 200 nm are typically more rapidly uptaken and sequestered from the blood circulation by resident immune cells, such as the Kupffer cells in the liver and the splenic macrophages, following surface opsonization [22]. Also, the shape of the particles can influence the phagocytic activity of macrophages. Cylindrical or rod-shaped NPs can avoid phagocytosis compared with spherical NPs with the same volume [23,24]. All of these aspects can be exploited for targeting the diseased vasculature.

The standard approach for designing nanoparticles has been formulation-driven, in which the payload’s physicochemical properties dictate the carrier material and the formulation process. This strategy allows that therapeutics with similar characteristics could be incorporated in the same nanocarrier. Remote loading has also been largely used to entrap amphiphilic and ionizable drugs, such as doxorubicin, in liposomes [25]. Alternatively, the drug molecules can be re-designed to match and improve the nanocarrier's properties. For example, chemical modification of drugs, such as doxorubicin or docetaxel, was able to modify the hydrophobicity and miscibility of poly(lactic-co-glycolic acid) (PLGA) nanoparticles [[26], [27], [28]].

The main classes of nanomaterials that have been used in CVD are micelles, liposomes, polymeric nanoparticles, dendrimers, carbon nanotubes, and inorganic NPs, most commonly formed from crystalline metals such as gold, silver or silica [13,29].

**Fig. 1:**
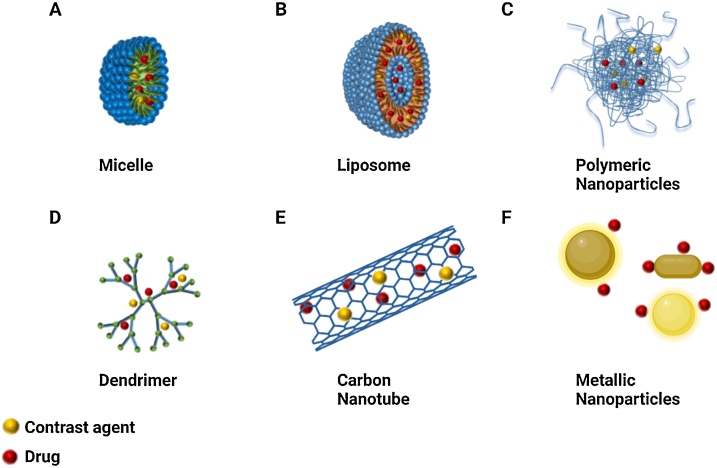
**Schematic representation of nanoparticles for theranostics.** (A) Micelle; (B) Liposome; (C) Polymeric nanoparticles; (D) Dendrimer; (E) Carbon nanotubes; (F) Metallic nanoparticles. Figure created in BioRender (https://biorender.com/).

Micelles (Fig. 1A) are spherical amphiphilic structures characterized by a hydrophobic core and a hydrophilic shell, allowing the delivery of poorly water-soluble drugs. They are formed by the self-assembly of amphiphilic molecules. Liposomes (Fig. 1B) are spherical nanoparticles characterized by one or more lipid bilayers consisting of single amphiphilic lipids or different lipids either charged or neutral. The phospholipids form a bilayer with an aqueous core. They can entrap several therapeutic molecules (vaccines, genetic material, biomolecules, and drugs). This type of nanoparticles is the most used/tested nano-drug delivery system (DDS) in basic and clinical medicine approved by the Food and Drug Administration (FDA).

Polymeric nanoparticles (Fig. 1C) can be realized using a variety of materials, including hydrophilic and hydrophobic polymers, synthetic and natural polymers, and combinations thereof. This platform usually presents a spherical-like structure with a size ranging between 50 and 250 nm. The choice of the polymeric materials is often related to the physico-chemical properties of the payload and expected release profiles. After their approval as materials for surgical sutures, poly(lactic acid) or poly(lactide) (PLA), and poly(lactic-co-glycolic acid) or poly(lactide-co-glycolide) (PLGA) became the materials for the synthesis of delivery drug system, thanks to their degradability in the environment and biocompatibility [30]. Also, the different molecular weight, molecular weight distribution and composition of these polymers allow easy tuning of the size, shape and biodegradability of the NP, influencing the pharmacokinetics of the loaded hydrophilic and hydrophobic therapeutic agents [31].

Dendrimers (Fig. 1D) are hyperbranched polymeric macromolecules with unique, well-defined three-dimensional architecture. In general, their growth starts from the centre core and occurs in an outward direction by polymerization reactions. Due to the abundant terminal functional groups, they have the potential for carrying imaging agents and drugs.

Carbon nanotubes (Fig. 1E) are graphene sheets rolled into cylinders of nanoscale diameter and covered with spherical fullerene. Currently, carbon nanotubes have attracted interest for their good properties, such as flexibility interaction with the cargo, high stability, ability in the drug release in the target site, considerable strength. However, they are not biodegradable and are associated with toxicity [[31], [32], [33]]. Despite these negative properties, there are used for vaccine delivery [34], bioimaging [35], diagnostic application [36], drug delivery [37,38], gene delivery and gene therapy [[39], [40], [41]].

Metallic NPs (Fig. 1F) find application in both drug delivery and diagnosis. The most extensively metallic nanoparticles are iron, copper, gold and silver and, more recently, other metals have been exploited for NP generation, such as gadolinium, zinc oxide, titanium oxide, and selenium [42]. Only a few metallic NPs are in clinical use, while the majority are used in preclinical studies. They may possess properties like surface plasmon resonance (SPR) that other nanoparticles, like micelles or liposomes, do not have [42]. These properties can be exploited to track the interaction between NPs and their putative biological targets.

### 1.3 The requirement for NPs for cardiovascular theranostics

The mainstays of CVD diagnostic imaging within the clinic are small molecules such as radionucleotides in positron emission tomography (PET) imaging, iodinated compounds in computed tomography (CT) or gadolinium (Gd^3+^) chelates in magnetic resonance imaging (MRI). At the other end of the size spectrum are microbubbles, which can be as large as 8 μm and allow contrast-enhanced ultrasound of the vasculature. Small molecules have several important limitations for theranostic use, including their rapid elimination time, low avidity coupled with high toxicity and are generally constrained to a single imaging modality [14]. Microbubbles, owing to their large size, are restricted to circulation, so they are also limited in their ability to deliver therapeutic agents into extravascular compartments. NP-sized agents are much less commonly used clinically but are emerging as key pre-clinical research agents for diagnostic, theranostic and therapeutic applications [[43], [44], [45]]. NPs offer many advantages that make them prime candidates for theranostic use in CVD. These include: 1) the ability to carry a concentrated payload of a therapeutic drug to the target site, thus minimising adverse systemic effects; 2) can carry a high concentration of imaging tracer yielding higher sensitivity and specificity; 3) high (and multi-valent) avidity; 4) the ability to cross cell membranes and release drugs intracellularly; 5) versatile chemistry and composition to allow for simultaneous imaging and targeted drug release; 6) supporting a wide range of therapeutic strategies including drug delivery, photodynamic therapy (PDT) and photothermal therapy (PTT); 7) multi-modal imaging (the ability to be imaged by more than one imaging system); 8) combination of different optical or vibrational signatures for multiplexed imaging.

## 2 Nanoparticles for diagnostic imaging of CVD

The advantages just described that NP may bring to diagnostic/theranostic applications have led to extensive pre-clinical research on their potential to advance the capability of established medical imaging technologies such as PET and MRI. A number of ultra-small superparamagnetic iron oxide (USPIO) NPs were clinically approved as T2-weighted contrast agents for MRI (albeit not for CVD imaging), and despite a relatively good safety profile when compared with T1-weighted Gd^3+^ chelates, they failed to live up to initial expectations [13,46] and most have been withdrawn from the market except for limited use of Ferucarbotran (Resovist®) in some regions. Despite this setback, a number of in-human studies using USPIOs for diagnosing CVD have been reported, all of which rely on passive targeting of USPIOs to the plaque via a combination of EPR and macrophage phagocytosis. These studies have been comprehensively reviewed elsewhere [47,48]. To fully utilise the potential of NPs in diagnostic imaging will require targeting the NPs to specific molecules related to disease pathogenesis (molecular imaging). Endothelial adhesion molecules such as vascular cell adhesion molecule 1 (VCAM-1) and intercellular adhesion molecule-1 (ICAM-1) are attractive targets for diagnostic imaging since they are expressed on activated endothelial cells, the first hallmark of vascular dysfunction and inflammation, and their expression in atherosclerosis could facilitate early diagnosis. They also offer direct access to systemically administered agents due to their endothelial expression. In the case of VCAM-1 and ICAM-1, there is also intracellular uptake of NPs [[49], [50], [51]], allowing tissue accumulation of the contrast agent for stronger signal intensity. One study that took advantage of this cellular internalisation utilised a VCAM-1 targeted USPIO (VINP-1) which accumulated *in vivo* at sites of plaque formation in hyperlipidaemic apolipoprotein-E (apoE)^−/−^ mice, where it was predominately associated with VCAM-1 expressing endothelial cells and macrophages, allowing detection of early-stage atherosclerosis in young animals [52]. VCAM-1 targeted USPIOs have also been employed at higher field strengths (17.6 T), enabling higher resolution imaging of aortic root plaque formation in apoE^−/−^ mice [53]. USPIOs have also been targeted to macrophages as a means of measuring macrophage burden within the aorta of hypercholesteraemic rabbits [54]. Moreover, coating USPIOs in gold expands the functionalisation options of the particles due to promoting binding via thiol groups. The gold-coated USPIOs, targeting the macrophage marker CD163, were injected into apoE^−/−^ mice with significant detection by MRI at 48 h post-injection compared with control mice [55]. Gd^3+^ has also been incorporated into NPs for T1-weighted MRI. A Gd^3+^ containing micelle (P947) targeted against matrix metalloproteinases (MMPs) showed significantly higher contrast in atherosclerotic arteries *in vivo* compared with healthy vessels. Signal to noise ratio was also improved by the rapid clearance of the unbound agent [56]. Woodside et al. employed a Gd^3+^ containing liposome targeted to the α_4_β_1_ integrin with a high-affinity binding ligand (THI0567). Following injection of this compound, apoE^−/−^ mice were imaged at the clinically relevant field strength of 1 T, with the results showing high specificity for the tracer bound to plaque macrophages [57]. High-density lipoprotein (HDL) particles provide an amenable, natural platform for imaging atherosclerotic plaques as they can be readily modified to incorporate a variety of contrast agents for CT, fluorescence and MRI [58] with the incorporation of Gd^3+^ allowing targeting T1-weighted MRI imaging [59]. Moreover, oxidation of the apolipoprotein (apo) A‐I protein can yield HDL particles that show enhanced specificity for plaque macrophages [60].

^19^F based MRI contrast agents predominately include perfluorocarbons (PFCs), which have a long history of use in humans as blood replacement products and have better overall biocompatibility compared with USPIOs [61]. PFCs have been developed that target markers of inflammation within the endothelium of atherosclerotic mice such as VCAM-1 [62,63], as well as targeting thrombin for imaging thrombotic events [64]. MRI can also allow limited but potentially important (in terms of increasing diagnostic sensitivity) multiplexing by employing NP. An example of multiplexed imaged involved the use of ^19^F containing PFCs together with a Gd^3+^ containing elastin targeted probe which has been employed to measure inflammatory burden and fibrosis, respectively, in a mouse model of MI. This is made possible using a dual ^19^F/^1^H MRI coil with images acquired at the clinical field strength of 3-T [57]. An elastin-specific Gd^3+^ containing probe has also been combined with passively targeted USPIOs to predict fatal aneurysm rupture in the angiotensin-II mouse model. The combination of elevated T2-weighted signal from USPIOs, reflecting enhanced macrophage accumulation, combined with decreased T1-weighted signal from a reduction in elastin content provided a high degree of sensitivity and specificity in predicting death from abdominal aortic aneurysm (AAA) [65].

NP-based imaging has only sporadically been used in CT for imaging CVD and has involved the use of gold [66] or iodinated [67] NPs for targeting plaque macrophages; however, the low sensitivity of CT, which necessitates millimolar levels of contrast agent, makes translation to the clinic prohibitive. Nuclear imaging encompassing PET and single-photon emission computed tomography (SPECT) predominately utilise small molecule tracers although sulphur colloids labelled with ^99^m-technetium (^99m^Tc) are commonly used for SPECT clinical imaging of various tissues including spleen, liver and bone marrow [68]; however, they are not used for imaging the cardiovascular system. Pre-clinical research has shown that ^64^Cu-tagged NPs can successfully be used for molecular imaging in the vasculature via PET with NPs targeted to both C-C chemokine receptor type 5 (CCR5) and receptor for advanced glycation end-products (RAGE) being employed in mouse models of vascular injury and ischaemia, respectively [69,70]. Notably, PET is also the first modality to be trialled in CVD patients in association with a targeted NP, in this case, a copolymer-based, natriuretic peptide receptor C (NPRC)-targeted NP (^64^Cu-25 %-CANF-Comb). With NRPC being upregulated in atherosclerotic arteries, the goal is to determine if ^64^Cu-25 %-CANF-Comb can be used to provide significantly enhanced signal via PET/MR in carotid arteries where atheroma is present when compared to healthy arteries (ClinicalTrials.gov: Identifier: NCT02417688).

Emerging technologies also either require NP for signal generation or can employ NP for enhanced sensitivity and imaging depth. Photoacoustic imaging (PAI) is one such example. PAI utilises short-wavelength optical pulses to generate acoustic energy of native molecules or external tracers that can be detected by US transducers. The use of multiple excitation wavelengths, as applied using multispectral optoacoustic tomography (MSOT), allows multiplex detection of either distinct biological molecules or external imaging agents, which can be tuned to have narrows absorption spectra, thus facilitating separation of the different tracers. MSOT has shown proof of concept in non-invasively imaging the human carotid artery [71] and is currently being trialled for assessment of atherosclerotic plaques in CVD patients (ClinicalTrials.gov: Identifier: NCT04237064). To unleash the full potential of PAI/MSOT, researchers have developed a variety of both organic and inorganic NPs compatible with PAI [72]. VCAM-1 targeted gold-based NPs have been employed for imaging atherosclerotic vessels [73,74]. To reduce interference from biological tissues and improve signal to noise, some researchers have moved from imaging in the near-infrared window to the ‘second’ or ‘far’ infrared range (1000−1700 nm) [75]. Utilising a 50 nm copolymer CD36-targeted NP in conjunction with excitation at 1064 nm, Xie et al. [76] demonstrated that photoacoustic signal enhancement was observed in carotid arteries of mice with atherosclerotic plaques. The signal enhancement was specific to CD36-targeted NP indicating the potential for PAI to detect macrophage accumulation within atherosclerotic lesions.

While NPs may be considered optional imaging agents for the modalities discussed thus far, other emerging technologies have a fundamental requirement for NP to generate a signal. One such example is magnetic particle imaging (MPI), which directly detects metallic NPs, generating a positive contrast image [77]. The detection of magnetic iron oxide-based NP draws parallels with MRI but offers superior sensitivity and signal to noise due to the lack of background interference [78]. In this regard, it is functionally closer to nuclear imaging than MRI. MPI is still at the preclinical stage with studies designed to demonstrate proof of concept across a variety of disease models. Utilising myeloperoxidase (MPO)-specific SPION based NP, Tong et al. [79] recently demonstrated the ability of MPI to detect macrophage-associated MPO activity within atherosclerotic plaques in apoE^−/−^ mice with the greatest signal derived from plaques showing hallmark features of instability [79]. While MPI has limitations such as lack of anatomic information, thus requiring hybrid MPI/MRI or MPI/CT imaging, the ability to derive quantitative data at a higher sensitivity than MRI, combined with NP that have a long-established use in human MRI imaging, makes MPI a promising technology for clinical translation.

Finally, surface-enhanced Raman spectroscopy (SERS) has been used in conjunction with gold NP targeted to adhesion molecules on activated macrophages [80] and *in vivo* blood vessels within the ear pinna of mice subjected to local inflammatory challenge [81]. The multiplexing potential of SERS has also been described in an *in vivo* model involving transplantation of human adipose tissue into immunocompromised mice [82,83]. Following injection of human TNF-α to upregulate inflammatory molecules on the surface of the human vessels within the engrafted tissue, SERS gold-NP (targeted to either VCAM-1, ICAM-1 or P-selectin) were simultaneously injected, and SERS spectra obtained from non-invasive imaging over the graft region. The narrow band emission spectra from each of the uniquely associated Raman reporter molecules could be separated from the multiplex signal to provide a quantitative analysis of the respective magnitude of NP binding which in turn correlates with adhesion molecule expression [82].

## 3 Nanoparticles as theranostics in CVD

As discussed, theranostics is the combination of diagnostic and therapeutic capability on a single agent. It is particularly important in the context of being able to track drug delivery and/or monitor the therapeutic efficacy of the delivered drug (such as the thrombolytic rate or decreased immune cell accumulation). As mentioned in section 1.2, NPs have can be loaded with a therapeutic agent and molecule (or molecules) that also allow diagnostic/theranostic imaging. This is a significant advantage over small molecules or molecular probes. MRI has been employed in combination with paramagnetic, Gd^3+^ incorporated and ^19^F based NP for theranostic research (Table 1). The use of α_v_β_3_ integrin-targeted paramagnetic NPs loaded with the anti-angiogenic compound fumagillin can effectively reverse angiogenesis in the aortic adventitia of atherosclerotic rabbits, with the extent of neovascularisation being monitored by MRI [84,85]. Yu et al. [86] developed Gd^3+^ incorporated NP targeted to extra domain B of fibronectin (FN-EDB), a splice variant of fibronectin that is upregulated in both human and murine atherosclerotic plaque. Following intravenous injection into apoE^−/−^ mice, FN-EDB targeted NPs were readily detectable in the brachiocephalic artery, with uptake correlating with FN-EDB expression. Moreover, injection of FN-EDB treated NPs loaded with cyanine (acting as a model drug) resulted in 6.9 times higher accumulation of cyanine within the plaque compared to the free drug. The NP preparation also bound to isolated human atherosclerotic arteries suggesting FN-EDG may be a viable and highly specific marker for atherosclerotic plaque imaging in patients [86]. Gd^3+^ has also been incorporated into latex-based lipid-coated NPs (LiLa), where the lipid coating is designed to target phagocytic macrophages within atherosclerotic plaques. The hydrophobic core is amenable to loading with a therapeutic agent that is released upon phospholipid layer degradation following cellular uptake. Gd-Lila NPs were uptaken by M1 macrophages *in vitro* and *in vivo* and showed preferential homing to macrophages in the atherosclerotic aorta with effective delivery of the model drug, rosiglitazone [87]. The use of NPs with lipid derivates that activate macrophage scavenger receptors may also prove a useful tool for directing NPs towards sites of vascular pathology avoiding systemic side effects. Solid lipid NPs that contain a core of the iron oxide, maghemite, surrounded by a lipid shell were developed by Oumzil et al. [88]. These NP are amenable for MRI imaging and following loading with prostacyclin (PGI2), effectively inhibited platelet aggregation in human blood samples [88].

**Table 1:** Examples of the theranostic use of nanomaterials in CVD.

Target	Nanoparticle	Imaging modality	Therapeutic	Result	Ref.
α_v_β_3_ integrin	paramagnetic	MRI	Fumagillin	Anti-angiogenic effect in aortic adventitia of atherosclerotic rabbits that could be monitored by MRI	[84,85]
FN-EDB	APT_FN-EDB_-[Gd]NP	MRI	Model drug only	NP bound to both isolated human atherosclerotic vessels and mouse vessels *in vivo*. NP carried model drug showed superior plaque accumulation versus free drug	[86]
Macrophage scavenger receptors	Lipid-latex hybrid (LiLa)	MRI, fluorescence	Rosiglitazone	Uptake by M1 macrophages allows MRI imaging and drug delivery upon intracellular NP degradation	[87]
Platelets	Iron oxide containing solid lipid NP	MRI	PGI2	Reduction of platelet aggregation in human blood samples	[88]
Infarcted heart	^18^F-rhodamine 6G and iron oxide NP labelled mitochondria	MRI, PET	Mitochondria	Following intracoronary perfusion of mitochondria entered into infarcted rabbit heart as shown by PET/MRI imaging and reduced infarct size and improved cardiac function	[90]
Cardiomyocytes and stem cells	Iron oxide-based NP (MagBICE)	MRI	Stem cells	Dual-targeted NP, either with or without magnetic targeting enriched stem cells into heart and reduced cardiac damage following MI	[91]
Macrophage scavenger receptors	HDL-MNS	MRI	HDL	5 times increase in T2-weighted MRI contrast compared with Feromoxytol and increase in macrophage cholesterol efflux	[93]
Macrophage scavenger receptors	PLGA-HDL	NIRF	HDL	Accumulation in atherosclerotic aorta and enhanced cholesterol efflux from macrophages	[94]
Thrombin	PFCs	MRI	PPACK	Inhibition of thrombosis at sites of acute thrombotic injury due to binding/uptake	[64]
Passive accumulation via EPR	L-PLP	MRI, ^18^F-FDG-PET/CT	Prednisolone phosphate (PLP)	NP entered atherosclerotic lesions with PLP inducing localised anti-angiogenic and anti-inflammatory effects as measured by MRI and PET Uptake of l-PLP confirmed in human patients with atherosclerosis but no change in either angiogenesis or inflammation was observed	[95] [97]
Passive	Hydroxybenzyl alcohol (HBA)-incorporating copolyoxalate (HPOX) Copolymer	NIR fluorescence	H_2_O_2_ scavenging, 4-AN	Intrinsic antioxidant effect in a mouse model of I/R injury. NP were formulated with the chemiluminescent sensor rubrene for NIR imaging and 4-AN as model drug	[98]
VCAM-1, macrophages and fibrin	Simian virus 40 (SV40) based NP	NIR fluorescence	Hirulog	Effective targeting to atherosclerotic plaques in mice, *in vivo* NIR imaging and delivery of hirulog, a thrombolytic drug	[99]
ROS	Macrophage-targeted theranostic nanoparticles (MacTNP)	NIR fluorescence	Photodynamic therapy	Accumulate inside macrophages *in vitro* and induce cell death via light exposure	[101]
Dectin-1	Glu/Ce6 nanocomplexes	NIR fluorescence	Photodynamic therapy	Accumulate inside macrophages *in vitro* and induce cell death via light exposure	[102]
Scavenger receptor-A (SR-A)	Ce6/DS-DOCA nanoagents	NIR fluorescence	Photodynamic therapy	Accumulate inside macrophages *in vitro* and induce cell death via light exposure	[103]
Passive (phagocytosis)	Gold nanorods	CT	Photothermal toxicity	Taken up by macrophages at sites of vascular injury. Following application of NIR light, macrophages were killed via photothermal toxicity	[104]
Passive	Silica-gold nanorods	Intravascular photoacoustic/ultrasound	Photothermal toxicity	Simultaneous induction and monitoring of photothermal excitation in isolated human coronary artery	[106]
Passive	Gold nanostars	SERS	Mitoxantrone	Gold nanostars show preferential accumulation in the heart following intravenous injection in mice, which can be visualised by SERS	[108]

MRI compatible NPs have also been tested in animal models of ischaemia/reperfusion (I/R) injury. Following the onset of ischaemia and extending through the reperfusion phase of I/R injury, cardiomyocytes undergo cell damage and apoptosis. Previously, the administration of autologous respiration-competent mitochondria has shown promise to replace damaged mitochondria in compromised cardiomyocytes in isolated rabbit hearts subjected to I/R injury [89]. Expanding on this research, Cowan et al. [90] generated ^18^F-rhodamine 6G and iron oxide NP labelled mitochondria and performed intracoronary infusion in Langendorff-perfused rabbit hearts subjected to I/R. PET and MRI imaging revealed that injected mitochondria entered the heart and remained there throughout the period of reperfusion. Moreover, there was a significant reduction in infarct size observed in the group that received the mitochondria injection [90]. Cheng et al. [91] developed a dual antibody-conjugated iron oxide-based NP (MagBICE) for use in MI. The NPs were functionalised with antibodies against the myosin light chain (for cardiomyocyte binding) and either CD45 (for exogenous bone marrow-derived stem cell binding) or CD34 (for endogenous stem cell binding). The NPs were able to enrich stem cells within the infarcted heart in a rat model of MI, a process that could be enhanced through targeting with an external magnetic field. The enhanced infiltration of stem cells induced by MagBICE resulted in greater retention of cardiac function and a reduction in cardiomyocyte loss. Thus, MagBICE NP brings together imaging and a multifunctional magnetic-molecular targeting system in one entity [91] (Table 1).

As discussed in section 2, HDL is an attractive vehicle for targeted diagnostic imaging in atherosclerosis owing to its affinity for plaque macrophages. Its endogenous function of inducing reverse cholesterol transport in macrophages [92] and thus removing excess cholesterol from the vessel wall also bestows it with a potential plaque regressing effect. By utilising high-density lipoprotein-like magnetic nanostructures (HDL-MNS), Nandwana et al. [93] demonstrated that superior contrast enhancement was observed when imaging macrophages when compared with Ferumoxytol. Moreover, the uptake of HDL-MNS resulted in an increase in cholesterol efflux [93] suggesting a possible dual imaging/therapeutic function for these biocompatible constructs. HDL-mimetic NPs have also been devised, which contain a PLGA core, which may facilitate rate-controlled drug release [94]. By creating PLGA-HDL NP that contained the fluorescence dye DiR (1,1′-dioctadecyl-3,3,3′,3′-tetramethylindotricarbocyanine iodide), it could be observed via fluorescence microscopy that the NP accumulated within plaque macrophages throughout the aorta in apoE^−/−^ mice [94]. PFCs have been functionalised with PPACK, a thrombin inhibitor that allows effective targeting to areas of vascular damage and inhibition of clot formation. The combination of ^19^F and ^1^H MRI allows NP binding and clot dissolution to be monitored, respectively [64]. Lobatta et al. [95] developed a liposomal preparation that encapsulated the glucocorticoid prednisolone phosphate (PLP). By incorporating Gd^3+^ into a liposome, T1-weighted MRI could be performed before and after administration to track liposome (L-PLP) accumulation within the aortic plaque of atherosclerotic rabbits. Moreover, by comparing images pre- and post- liposome delivery using dynamic contrast-enhanced MRI (DCE-MRI), the authors were able to determine changes in plaque angiogenesis. These therapeutic-derived changes also correlated with local reductions in inflammation as determined by ^18^F-FDG PET/CT [95]. This is an elegant example of multi-modal theranostics in which a NP formulation can be utilised in conjunction with diagnostic imaging to both deliver/track a therapeutic agent and subsequently monitor the cellular and morphological changes that result following drug release. It was later demonstrated that in the rabbit model of atherosclerosis, liposomes accumulate in the plaque via the EPR effect, entering from both the expanded vasa vasorum and through disrupted endothelium on the luminal side [96]. The EPR effect within human atheroma may be greater still due to the greater adventitial blood supply, and hence passive targeting of NP may be a viable mechanism of delivering therapeutic agents to atherosclerotic plaques without the potential concerns around active targeting such as agonistic actions of targeting ligands or other unwanted pharmacodynamic effects. To understand how PLP loaded liposomes may affect human atherosclerosis, a randomized, placebo-controlled, double-blinded trial of 30 patients with atherosclerosis was performed [97]. ^18^F-FDG PET/CT was performed prior to treatment to confirm the presence of active inflammation within the carotid artery wall. Similar to the aforementioned study in rabbits [95], DCE-MRI and ^18^F-FDG PET/CT were performed before and after liposome NP treatment. Disappointingly, however, in contrast to the pre-clinical model, no change in angiogenesis or inflammation was observed, despite successful trafficking of the NP to plaque macrophages. The authors hypothesised that insufficient dose or duration of treatment may have explained the lack of therapeutic effect [97] (Table 1).

NPs that contain fluorescent molecules or are designed to react with endogenous biomolecules to create fluorescent products have been developed for use with near-infrared (NIR) imaging and can be modified to produce a therapeutic effect (Table 1). Lee et al. [98] developed a NP composed of a hydrogen peroxide (H_2_O_2_)-responsive polymer. The H_2_O_2_ scavenging properties of this NP endows it with an intrinsic therapeutic effect. Moreover, upon the incorporation of the chemiluminescent sensor molecule, rubrene, the magnitude of the H_2_O_2_ reaction could be monitored in the mouse hind limb model of I/R injury. The theranostic use of this NP was extended still further by loading the NP with the PARP-1 inhibitor and the anti-apoptotic drug, 4-amino-1,8-napthalimide (4-AN), which reduced local apoptosis following I/R injury [98].

Quantum dots (QD) are bright, small (<10 nm) fluorescent particles formed from an encapsulated semiconductor metallic core [14]. In another example of NIR theranostics, Sun et al. [99] utilised a simian virus 40 (SV40) based NP system, containing the NIR excitable QD, QD800 with 3 formulations targeted to either VCAM-1, macrophages or fibrin. Both *in vivo* and *ex vivo* imaging of apoE^−/−^ mice showed accumulation of SV40 particles and NIR fluorescence at areas of atherosclerotic plaque formation. Multifunctional SV40-based NP that also contained the small anti-thrombin peptide inhibitor, hirulog, were targeted to macrophages and were shown to effectively inhibit thrombin at sites of arterial accumulation [99].

Photodynamic therapy (PDT) involves the combination of a photosensitising agent and external light to induce the killing of cells, via increased production of ROS such as singlet oxygen [100]. NPs of various compositions have been designed to target inflammatory macrophages via ROS [101], dectin-1 [102] and scavenger receptor-A (SR-A) [103] *in vitro* where upon uptake and exposure to light irradiation, induce cell death. This ‘double hit’ mechanism can allow a highly selective killing of macrophages, without damaging neighbouring cells (Table 1).

Conductive NP (e.g. metallic or carbon-based) have also been used in combination with laser excitation to induce photothermal heating and phototoxicity of cells involved in disease processes. This field has gained traction in oncology but is also an attractive avenue of research in CVD, where the aberrant function of inflammatory monocytes and macrophages coupled with their high phagocytic rate makes them a prime candidate for this approach. NP can be modified by size and material to optimise the photothermal effect and to be used with a variety of imaging tools. Gold nanorods are readily taken up by macrophages *in vitro* and *in vivo* at sites of arterial injury and, following excitation with NIR light, undergo photothermal heating resulting in localised killing of macrophages [104] (Table 1). Single-walled carbon nanotubes are also phagocytosed by macrophages and can be effective at mediating phototoxicity of macrophages within sites of arterial injury [105]. While photothermal therapy with exogenous NP offers a high degree of specificity owing to phototoxicity only occurring in cells that accumulate NPs, significant hurdles remain with regards to clinical translation, with the primary limitation being the low penetrance of NIR, which may necessitate the use of invasive catheter-based devices. To this end, a catheter that combines intravascular photoacoustic imaging (IPAI) with intravascular ultrasound (IVUS) has been tested on isolated human coronary arteries as a means to simultaneously detect local accumulation of gold nanorods while also inducing/monitoring the photothermal effect [106]. Despite these limitations, photothermal therapy has been successfully trialled in human patients with coronary artery disease. Following intracoronary injection of iron-bearing silica-gold NPs, targeted to CD68 bearing macrophages, transcutaneous NIR was applied via a high-power laser resulting in ‘detonation’ of the NPs and plaque shrinkage. Notably, no adverse events were reported in the 12 months follow-up [107]. Finally, gold nanostars conjugated to the anti-cancer drug mitoxantrone (MTX) were shown to accumulate in the healthy heart of mice and could be imaged *in vivo* by SERS [108]. These MTX-nanostars were shown to cross the endothelium and enter cells within the heart and aorta just 5 min after intravenous injection [108]. Thus, gold nanostars in combination with SERS could offer a novel theranostic strategy for tracking and delivering therapeutics to the vasculature and myocardium (Table 1).

## 4 Future perspective

Nanomedicine and nano-delivery are rapidly developing. Materials, which are in a nanoscale range, are used as diagnostic tools or deliver therapeutic agents to specifically targeted sites in a controlled manner.

Nanomedicine holds the potential to revolutionize diagnostics and the therapeutic approach in several conditions from cancer to CVD, including neurological and infectious diseases. The inherent ability of nanomedicines to enhance drug bioavailability and cell-specific accumulation can only boost the balance between their efficacy and toxicity [109] - therapeutic index.

Despite the approval of several nanomedicine anticancer drugs, such as Onivyde (liposomal irinotecan) and Vyxeos (liposomal daunorubicin plus cytarabine), or even most recently BNT162b2 (Pfizer-BioNTech) or mRNA-1273 (Moderna), the success rate of clinical translation remains relatively low. The success of BNT162b2 and mRNA-1273 represents an incredible effort in the mitigation of the COVID-19 global health crisis of unprecedented dimensions in modern history. This is indeed a clear demonstration of how impactful the application of nanomedicine can be in different clinical contexts. This is becoming the focus of an intense debate about the small number of nanomedicine and theranostic products approved against the increasing number of preclinical studies based on nanomedicines.

Translating nanomedicines from preclinical studies to the clinical side requires smart thinking and rational and realistic reasoning and, equally importantly, infrastructures dedicated to clinical-volume fabrication under good manufacturing practices. In the next few years, the biotechnology and pharmaceutical technology space should invest more time and resources in the development of innovative modular diagnostic and drug delivery systems, combining different materials, targeting moieties, therapeutic agents (synthetic small molecules, antibodies, peptides, oligonucleotides) and, at the same time, identify more efficient manufacturing processes under the overarching goal of accelerating the clinical integration of nanomedicine and its application in theranostics.

## Funding sources

Our work was supported by the British Heart Foundation (BHF) [grant PG/19/84/34771, PG/21/10541]; the Engineering and Physical Sciences Research Council (EPSRC) [grant EP/L014165/1]; the 10.13039/501100000781European Research Council [Project Identiﬁer: 726318]; the 10.13039/100010269Wellcome Trust [grant 204820/Z/16/Z]; and the University of Glasgow Scottish Funding Council and the Global Challenges Research Fund.
